# Bacterial two-hybrid systems evolved: innovations for protein-protein interaction research

**DOI:** 10.1128/jb.00129-25

**Published:** 2025-08-01

**Authors:** Rebecca M. Richardson, Steven M. Pascal

**Affiliations:** 1 Department of Chemistry and Biochemistry, Old Dominion University 733982, Norfolk, Virginia, USA; Geisel School of Medicine at Dartmouth, Hanover, New Hampshire, USA

**Keywords:** protein–protein interactions, bacterial two-hybrid, two-hybrid screening

## Abstract

Bacterial two-hybrid (B2H) systems offer a versatile platform for detecting protein-protein interactions (PPIs) *in vivo*. Originally developed to study bacterial transcriptional regulators, these systems have evolved to support a wide range of applications, including interaction mapping, domain analysis, and high-throughput screening. This review surveys the development and expansion of B2H systems across diverse biological contexts. We highlight technical considerations, platform-specific innovations, and recent adaptations that extend the utility of B2H systems in both prokaryotic and eukaryotic protein interaction research environments. By comparing different B2H variants and discussing their integration with complementary approaches, we provide a framework for leveraging these systems in modern PPI discovery.

## INTRODUCTION

Protein-protein interactions (PPIs) are fundamental to nearly all biological processes, governing cellular signaling, enzymatic activity, structural integrity, and regulatory mechanisms. Efforts to manipulate these interactions have driven advances in therapeutics and biotechnology (1). Given that over 80% of proteins function within complexes rather than in isolation (2, 3), mapping and understanding PPIs is critical for elucidating cellular function and disease pathology.

The complexity of protein interactomes arises from their dynamic nature—PPIs are influenced by factors such as post-translational modifications (PTMs), spatial and temporal regulation, and environmental conditions (4, 5). These interactions play essential roles in cellular pathways, affecting enzymatic activity, protein specificity, and signal transduction. The ability to accurately model and analyze these networks has broad applications, from mapping essential protein pathways to identifying novel drug targets (3–6). However, the study of PPIs presents significant challenges, including technical limitations, resource accessibility, and the need for combinatorial approaches to fully capture the scope of interaction networks (1, 2, 4–6).

A variety of techniques have been developed to detect and characterize PPIs. Many of these methods require specialized equipment, extensive optimization, and significant resources, limiting their accessibility (1, 2, 6). However, two-hybrid assays—particularly bacterial two-hybrid (B2H) systems—offer a cost-effective and scalable approach to study PPIs using standard molecular biology techniques, making them an attractive tool for initial screening and validation of PPIs. Despite these advantages, the potential of B2H systems remains largely untapped, particularly in comparison to the more widely adopted yeast two-hybrid (Y2H) system.

This review explores the variety of available B2H assays, highlighting their strengths, limitations, and potential for integration with other approaches to advance our understanding of protein interaction networks. By surveying these developments, we aim to provide a comprehensive view of B2H system capabilities across a diverse range of experimental settings.

### Two-hybrid assays

The development of two-hybrid assays stemmed from foundational discoveries in gene regulation and protein modularity. Seminal findings in the 1960s, such as Jacob and Monod’s operon model in bacteria, demonstrated that proteins regulate transcription by interacting with specific DNA sequences (7). By the 1980s, researchers such as Keegan et al. (8) had shown that eukaryotic transcription factors (TFs) were composed of modular domains (8). This discovery was pivotal, as it established that these domains could function independently and be reconstituted artificially (8). Building on this concept, in 1989, Fields and Song hypothesized that two proteins of interest (POIs) could be fused to these modular domains, and if the proteins interacted, they would reconstitute a functional TF (9).

The GAL4 TF of *Saccharomyces cerevisiae* consists of two independent domains: a DNA-binding domain (DBD) that targets a particular gene, and an activation domain (AD) that interacts with transcription machinery to enhance transcription of a reporter gene (8, 9). In a Y2H assay, two separate POIs are fused to these two domains. If the proteins interact, their physical association will bring the DBD and AD into close enough proximity to reconstitute a functional TF. This restored activator drives the expression of a reporter gene, such as *lacZ*, allowing the detection of interactions through selective growth on nutrient-deficient media or via enzymatic colorimetric assays (9). The development of Y2H resulted in a scalable *in vivo* alternative to traditional biochemical methods, such as co-immunoprecipitation (co-IP) and affinity chromatography (9, 10). The introduction of additional reporter gene systems further enhanced the method, enabling quantitative detection of PPIs. This innovation laid the groundwork for a wide range of two-hybrid systems that are still in use today, revolutionizing proteomics research (11).

This *LacZ*-based Y2H system has been widely employed due to its scalability and ease of use, particularly in high-throughput (HTP) screens for large-scale interactome mapping (2, 5, 11, 12). However, users are faced with challenges such as high false-positive rates and limited applicability to membrane proteins or interactions requiring specific subcellular environments (2, 11). To address these limitations, several variants have emerged. For example, the membrane Y2H system allows the study of membrane-bound proteins by utilizing split-ubiquitin technology (2, 6). Similarly, mammalian two-hybrid (MaMTH) systems offer a more physiologically relevant context for examining human PPIs (11). Complementary methods have also gained prominence alongside Y2H, including affinity purification-mass spectrometry (AP-MS) and proximity labeling techniques. These approaches enhance the detection of transient or weak interactions, providing a more comprehensive view of cellular interactomes (1, 3, 5).

### The emergence of B2H systems

**Fig 1:**
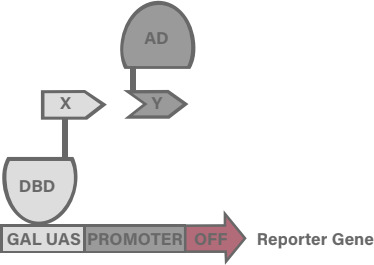
Schematic diagram of two-hybrid systems. Two-hybrid systems regulate reporter gene expression through interactions between fused protein domains. In the example presented in this Figure, the proteins to be assayed for interaction (X and Y) are fused, respectively, to the DBD and the AD of a split TF. Interaction between X and Y would effectively reconstitute the TF, which then activates transcription of the reporter gene.

B2H systems have emerged as powerful tools to address some of the specific limitations of Y2H. Y2H and B2H assays each typically detect PPIs by altering the expression level of a reporter gene through the interaction of a “bait” and “prey” protein (Fig. 1). However, the use of *Escherichia coli* as the host organism introduces faster growth, lower cost, and higher transformation efficiency relative to yeast (13–16). Moreover, B2H assays require only standard *E. coli*-based molecular biology techniques and expertise, making them easily accessible to more research laboratories. In addition, B2H systems can probe membrane protein interactions which may be challenging to analyze using Y2H (13, 14, 16–18). B2H systems can also overcome the inability of yeast cells to tolerate protein interactions that are toxic to eukaryotic cells or that otherwise require a bacterial environment (13–16).

PPI detection in B2H often relies on quantitative readouts such as β-galactosidase assays, which employ spectrophotometric measurements, or X-gal (5-bromo-4-chloro-3-indolyl-beta-D-galactopyranoside) assays which use qualitative blue-white colony screening (13–15, 19). Antibiotic resistance can serve as an alternative qualitative readout (13, 14, 18–20). These features make B2H systems suitable for HTP screening of cDNA or genomic libraries (14, 16).

B2H systems have been widely applied in functional genomics, mapping of protein interaction networks, characterization of binding interfaces through mutational analysis, and identification of small-molecule inhibitors of protein interactions (14, 18). As with all two-hybrid systems, they are susceptible to false positives and negatives, requiring secondary validation. Nevertheless, enhancements such as engineered transcriptional repressors and complementation strategies have improved specificity and broadened applicability (14, 18).

B2H systems have been primarily used for studying bacterial PPIs. However, they have also been successfully applied to non-bacterial interactions, including mammalian PPIs (15, 16, 21). Using a bacterial host to study eukaryotic PPIs offers certain advantages, such as the absence of background eukaryotic proteins that could interfere with the assay (13, 15). In eukaryotic two-hybrid systems, endogenous host proteins can introduce non-specific interactions, leading to false positives or negatives that obscure the detection of PPIs. The interfering interaction could potentially result from a background protein that is similar to, or even identical to, one of the proteins being assayed. In contrast, bacterial systems offer a more controlled environment with reduced interference from host proteome complexity (13, 15, 22, 23). Differences in PTM systems of *E. coli* vs eukaryotic cells introduce a further challenge but also provide additional opportunities for complementary studies, as is discussed further in the next section.

Few studies have directly compared results from B2H and Y2H systems. Salinas et al. (24) conducted a mutational interaction analysis of the cyanobacteria PII-PipX-NtcA regulatory axis (24). They found that B2H detected several interactions missed by Y2H, including PipX-NtcA interactions mediated by residues Y32A, R35A, F38A, and R54C. These interactions, confirmed via Western blot and consistent with *in vivo* phenotypes in *Synechococcus elongatus*, suggest that Y2H overestimated the disruptive effect of these mutations (24). Moreover, B2H captured a “false positive” PipX self-interaction driven by indirect bridging through *E. coli* PII homologs GlnB and GlnK—an interaction not observed in Y2H assays. In these instances, B2H was better able to capture certain weak interactions (24). These observations suggest that a combination of approaches is likely to provide a more complete analysis.

Although B2H techniques are well established only with *E. coli*, the potential exists for expansion to alternative hosts. For instance, *Lactococcus lactis* and *Bacillus subtilis* offer translational and physiological features that may better support the expression of complex or membrane-associated eukaryotic proteins (25). *L. lactis* is less prone to forming insoluble protein aggregates and possesses a membrane lipid composition (cardiolipin and glycolipids) that supports the proper folding of membrane proteins (25). *B. subtilis* has well-characterized secretion pathways and chaperone systems which can assist in folding exported proteins (26, 27). Hosts with codon biases that better match the GC or AT content of a target gene may also enhance translation efficiency. For example, *Mycobacterium* species tend to be GC-rich, while *Mycoplasma* are AT-rich (28).

## B2H: GENERAL TECHNICAL CONSIDERATIONS

This section provides insight into technical considerations relevant to two-hybrid systems, with a specific focus on B2H. Two-hybrid systems, while invaluable, are not without limitations, and researchers must address several challenges to maximize their utility and reliability.

### Reporter gene selection and optimization

**Fig 2:**
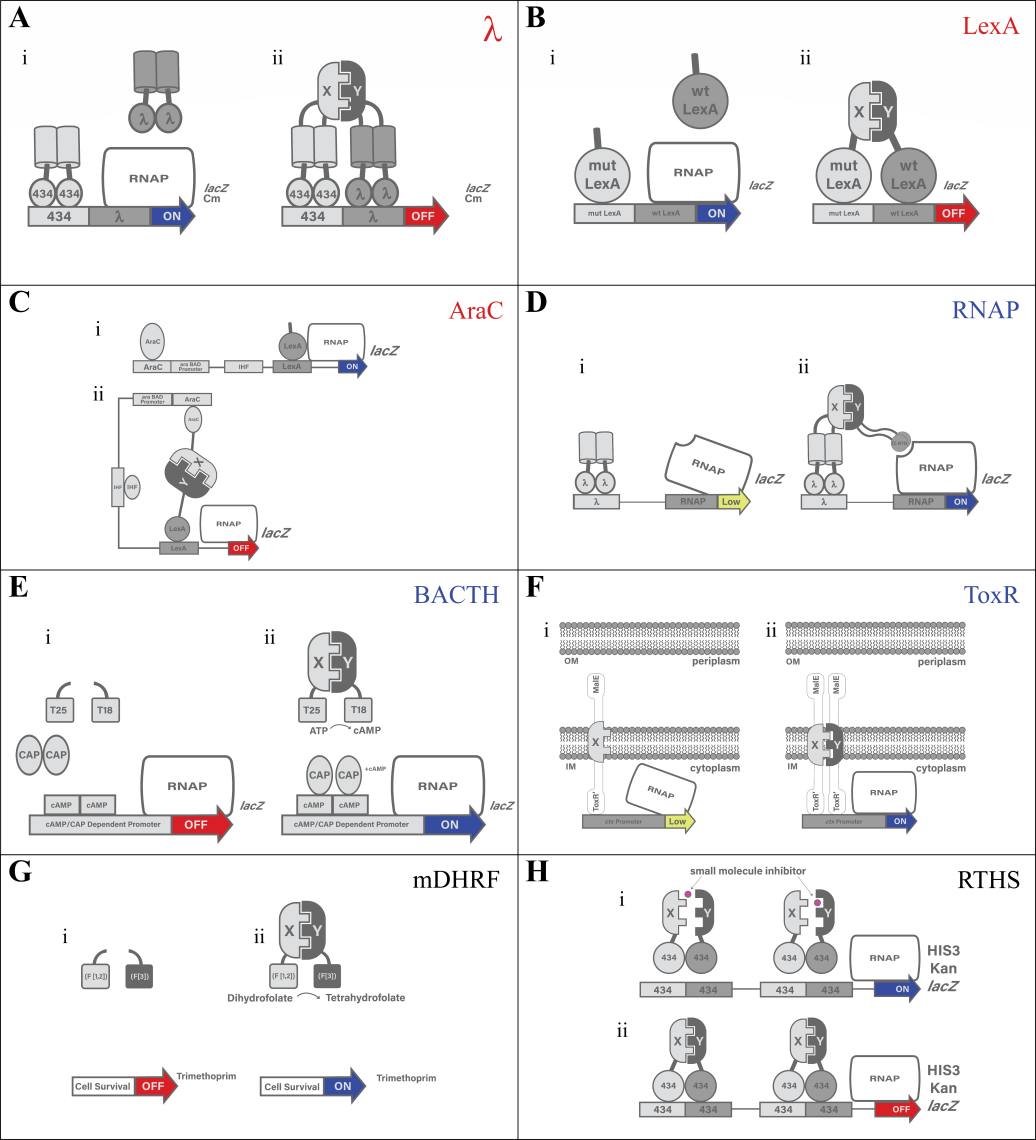
Overview of B2H systems. Schematic diagrams of the eight B2H variants presented in the section B2H variants: a systems overview. The name of the variant appears in the upper right-hand corner of each box. For each variant, the first diagram (i) depicts the absence, and the second diagram (ii) depicts the presence of the investigated PPI. With the exception of panel G, blue, red, and yellow arrows represent active, repressed, and low-level transcription, respectively. In panel G, red depicts cell death while blue depicts cell survival. Thus, variants A–C utilize transcriptional repression to detect the PPI, and the variant names are written in red font. Variants D–F utilize transcriptional activation or transcriptional enhancement to detect the PPI, and these variant names are written in blue font. Variant G and H both utilize cell survival to detect the PPI (although for variant H, the intermediary transcription control step is shown), and so these two variant names are written in black font. RNAP is RNA polymerase. A slanted RNAP (panels D and F) highlights low-level transcription and so occurs together with a yellow arrow. The other molecules and the detailed molecular mechanisms are described further in the text.

Reporter gene choice critically impacts B2H assay sensitivity, specificity, and quantification. Systems such as the bacterial adenylate cyclase two-hybrid (BACTH) (Fig. 2E) use cAMP-responsive backgrounds to broaden the dynamic range but require tightly regulated induction to ensure reproducibility (29). In ToxR-B2H (Fig. 2F), chloramphenicol acetyltransferase provides quantitative output but needs strict standardization to distinguish true interactions from background signal (30–32). Dual-reporter systems (e.g., HIS3 and *aadA*) require careful adjustment of selection pressure, such as 3-AT concentration, to avoid false negatives (33).

The modular nature of B2H platforms supports various reporters, including *lacZ* and fluorescent proteins, enabling both HTP screening and detailed mechanistic studies (14, 15, 34). However, reporter performance is influenced by promoter strength, fusion protein (FP) expression, and reporter-specific properties (13, 19, 35). As most B2H readouts rely on transcriptional activation, follow-up validation is essential to exclude indirect effects or artifacts from protein overexpression (13, 19).

### Reporter strain

The genetic background of *E. coli* influences B2H performance. Strains carrying regulatory elements such as *lacI*^*q*^ help suppress leaky expression of FPs, reducing background and toxicity from overexpression (33). This is critical when expressing cytotoxic partners such as membrane-associated or replication proteins, as seen in the reverse B2H system (RTHS) (Fig. 2H), where DnaC constructs were excluded due to host viability issues (36). In systems such as LexA (Fig. 2B), clear repression signals depend on the stability of heterodimers over homodimers, often requiring inducible promoters and low-copy vectors to prevent oversaturation (37, 38). Chromosomal integration can improve reproducibility by reducing copy number variation but makes operator or reporter modifications more difficult (38).

### Plasmid design and stability

Stable co-expression of bait and prey fusions requires careful plasmid selection. Low-copy vectors such as pBT and pTRG (used in RTHS, Fig. 2H) help reduce background expression but may yield lower material for downstream assays (33). Plasmid compatibility is also important; distinct origins of replication and non-overlapping antibiotic resistances prevent plasmid loss and ensure consistent expression across replicates (15, 39). FP design also affects interaction detection. Flexible linkers and orientation testing improve folding, solubility, and accessibility of binding interfaces (15, 19). Incorporating flexible linkers and testing alternative orientations of fusion tags can help maintain native protein function and reduce steric hindrance (15, 19). Streamlined cloning systems, such as Gillman et al.’s (40) Gateway-compatible method, simplify vector construction and allow rapid testing of multiple configurations without extensive subcloning (40).

### False positives and negatives

False positives may result from non-specific interactions or reporter auto-activation, while false negatives often stem from misfolding, aggregation, or assay incompatibility of FPs (4, 11). Inducible promoters that help to control expression levels can reduce folding stress (15, 19, 40, 41). Spatial and temporal mismatches between bacterial and mammalian systems also contribute to this challenge. Since bacteria lack organelle boundaries, proteins that would spatially segregate in eukaryotic cells can co-localize in the bacterial cytoplasm, increasing the chance of false positives (11, 42). Likewise, proteins that may not be simultaneously present in eukaryotic cells can interact in B2H (or Y2H) assays, since they are specifically induced during the assay.

B2H systems such as BACTH and ToxR (Fig. 2E and F) improve detection of membrane-associated PPIs but still face challenges with protein insertion and tag orientation (43, 44). Misfolding and aggregation are more common with large, multi-domain proteins, as *E. coli* lacks specialized eukaryotic chaperones (45). Misfolded intermediates can expose hydrophobic surfaces that promote aggregation, resulting in insoluble inclusion bodies during bacterial expression (46). This can obscure true interactions. Co-expression of chaperones can sometimes improve solubility and rescue signals (46–48). Difficulties in detecting interactions involving membrane-bound or toxic proteins can be exacerbated by the properties of the host organism or the intrinsic nature of the proteins being studied (11, 24). Although BACTH (Fig. 2E) has been adapted for disulfide-dependent and membrane PPIs (30, 49, 50), cross-reactivity with host proteins remains a concern. Rigorous fusion design and appropriate negative controls are essential to minimize artifacts (14, 51, 52).

### Quantification with B2H systems

Quantification in B2H systems remains a significant bottleneck, with most assays relying on reporters such as β-galactosidase (Table 2). In systems such as BACTH (Fig. 2E), *lacZ* expression reflects an indirect cascade (binding → cAMP → transcription → enzyme activity), providing qualitative output but lacking resolution to detect subtle affinity differences due to signal amplification and saturation effects (29, 43, 49, 53). Selection-based reporters (e.g., HIS3, antibiotic resistance) offer stringent screening but yield binary outputs unsuitable for affinity ranking (22, 54). Fluorescence-based reporters coupled with flow cytometry have improved sensitivity and dynamic range, enabling real-time, single-cell resolution and correlation with binding affinities across nanomolar to micromolar ranges (29, 39). Next-generation sequencing (NGS)-based methods such as next-generation B2H (NGB2H) use barcode ratios to infer interaction robustness and support HTP profiling, though direct mapping to K_d_ remains limited (29). Despite these advances, many B2H systems still depend on legacy reporters, underscoring the need for scalable, biophysically benchmarked quantitative readouts.

### Assay validation and controls

The potential for false positives and negatives makes the use of proper controls essential (4, 11, 24). Negative controls, such as individual bait or prey expression or pairing with non-interacting partners, help define background activity and set a baseline for non-specific interactions (14, 51, 52). Use of multiple reporter types can improve reliability by reducing dependence on a single readout (19, 39). Interactions identified in B2H should be validated with orthogonal methods such as co-IP, Förster resonance energy transfer (FRET), or isothermal titration calorimetry (ITC), which can confirm binding and provide data on affinity and specificity (4, 11). This multipronged validation is critical when B2H informs mechanistic studies, drug discovery, or domain mapping.

### PTMs

PTMs often regulate eukaryotic PPIs by altering protein folding, charge, and stability (41, 55–57). Some modifications inhibit binding; for example, O-GlcNAc glycosylation blocks the CREB-TAFII130 interaction, and phosphorylation alters PTEN conformation to disrupt PI3K-Akt signaling (56–58). Therefore, the PPIs should be evaluated under both modified and unmodified conditions when feasible (41).

While *E. coli* can perform limited PTMs such as phosphorylation, their frequency and complexity are far lower than in eukaryotes. Eukaryotic phosphorylation, primarily on serine, threonine, and tyrosine, is pervasive and central to cell signaling (59–62). In contrast, bacterial phosphorylation is mostly on histidine and aspartate and is responsive to environmental conditions (59–61). The reduced PTM background in B2H systems can aid detection of unmodified interactions that might be inaccessible in eukaryotic cells. Comparative screening using both PTM-limited (B2H) and PTM-permissive (e.g., Y2H, MaMTH) platforms helps identify modification-dependent interactions (22–24, 63). Interactions absent in B2H but present in eukaryotic systems suggest PTM reliance, while those found in both are likely independent of modification (64).

### Codon usage bias

Eukaryotic genes can often contain codons that are rare in *E. coli*, which can severely limit translation efficiency. *E. coli* tRNA pools are evolved to match *E. coli* codon frequencies; for example, clusters of rare codons (e.g., AGA/AGG for arginine or ATA for isoleucine) in a human open reading frame can deplete the corresponding tRNAs and stall ribosomes (65). This leads to truncated proteins or low-level expression (65). To address this, researchers frequently employ codon optimization, altering the DNA sequence of the eukaryotic gene to use synonymous codons preferred by *E. coli* (66). An alternative approach is to use *E. coli* strains engineered to supply extra tRNA copies for rare codons (42).

## B2H VARIANTS: A SYSTEMS OVERVIEW

Since their introduction, B2H systems have been modified and expanded to accommodate a variety of experimental needs, leading to the development of several distinct versions (Fig. 2; Table 1). Each adaptation introduces unique methodological refinements that enhance sensitivity, broaden the range of detectable interactions, or improve compatibility with specific proteins and PPI detection methods. The following sections review these B2H system variants, focusing on their unique features and method-specific technical considerations. Recent advancements and unique technical challenges are also presented. Collectively, these developments highlight the adaptability of B2H systems and their potential to play a pivotal role in molecular biology.

**TABLE 1:** Comparison of B2H systems

System	Innovation	Quantification method	Notes	Reference
Lambda bacteriophage two-hybrid (λ)	First B2H system. Foundational design links homodimerization to transcriptional repression. Addition of 434 allowed heterodimer detection.	β-Galactosidase assay	Cooperative repression	(67, 68)
LexA	Employs hybrid asymmetric operator with high/low-copy plasmids to better restrict transcription repression to heterodimeric PPIs.	β-Galactosidase assay	Repression-based, flexible vector usage	(38)
AraC	Uses DNA looping and operator spacing to control repression. DNA looping mechanism is tunable; unique spatial repression strategy.	β-Galactosidase assay	DNA looping mechanism; uses integration host factor-enhanced *E. coli* strains	(69)
RNA polymerase (RNAP)	PPI directly recruits RNA polymerase subunits to activate transcription. This increases sensitivity. Ideal for weak/transient PPIs.	β-Galactosidase assay	Direct RNAP recruitment; useful for weak/transient PPIs	(54)
BACTH	PPI reconstitutes adenylate cyclase activity; first B2H method not directly tied to transcription. Allows detection of PPI throughout the cytoplasm and membranes.	β-Galactosidase assay; cAMP measured by ELISA	Detects periplasmic and disulfide bond-dependent interactions	(49, 50)
Next generation	BACTH variation uses barcoded reporters and RNA-seq to quantify thousands of PPIs simultaneously. Uses next-gen sequencing for high-throughput quantitative PPI profiling.	Barcode RNA/DNA read ratio via NGS	Multiplexed PPI screening	(29)
ToxR	PPI drives ToxR dimerization in the bacterial cell membrane. Restricts PPI detection to integral membrane proteins in a native lipid environment.	β-Galactosidase assay	Validated for periplasmic interactions	(30)
Murine dihydrofolate reductase (mDHFR)	PPI reconstitutes murine DHRF activity; trimethoprim resistance-based selection; transcription-independent survival assay.	Bacterial survival in selective media (M9 + trimethoprim); growth rate linked to interaction strength	Enzymatic reconstitution; sensitive to linker design	(51, 52)
Reverse (RTHS)	Screens for disruptors of a specific PPI using survival as a readout when transcriptional repression is blocked.	Options include β-galactosidase assay, ELISA, fluorescence quenching	*In vivo* screen for PPI disruption; screens cyclic peptide libraries	(36)

### Variants using transcription repression

The following B2H variants utilize repression of a reporter gene to detect a PPI. These three variant names are colored red in Fig. 2.

### λ bacteriophage-based B2H (λ-B2H)

The λ-B2H system was first developed by Hu et al. (67) using λ cI repressor fusions to link homodimerization to transcriptional repression (13, 14, 18, 19, 67). A later variant by Hays et al. (19) incorporated both λ and 434 operators to detect heterodimeric interactions (19). For this review, the more widely adopted and referenced dual-operator configuration is used as the reference point for comparison with other B2H variants.

#### Basic setup

The λ-B2H system (Fig. 2A) utilizes cooperative binding between two repressors, λ cI and 434, to detect PPIs (19, 67, 68). In the ON state, these repressors bind independently but weakly to their respective operator sites, failing to fully inhibit transcription. The OFF state occurs when an interaction between two fused POIs (X and Y domains) brings λ cI and 434 repressors together, resulting in repression of transcription (19, 67, 68).

#### More detail

Both λ cI and 434 repressors are naturally dimeric DNA-binding proteins. This system exploits their need for cooperative binding to fully repress transcription (19, 67, 68). The λ cI repressor binds weakly to its low-affinity operator (λO_s_2). The 434 repressor functions similarly, but with distinct operator recognition for the 434 operator (O_con_). The system utilizes a hybrid operator combining recognition sites for each repressor, ensuring that only strong binding between the two repressors, via the fused POIs, leads to full transcriptional repression (19, 67, 68).

##### Example studies

Hu et al. (67) utilized site-directed mutagenesis and resistance to λ phage superinfection to identify mutations that disrupted hydrophobic interactions essential for dimer stability (67). This approach revealed the critical role of hydrophobic residues in stabilizing dimer interfaces in leucine zippers (LZ) and underscored the utility of B2H systems for studying complex interactions in controlled environments (19). Bunker et al. (68) used chimeric λ cI repressors to screen for interacting factors in cDNA libraries (68). Their method capitalized on the ability of dimerization domains to modulate transcriptional activity, providing a practical approach for identifying protein interactions. This innovation extended the utility of B2H systems beyond the study of LZ to a broad range of interaction motifs, facilitating the discovery of novel interacting partners and potential dominant-negative effects (19, 67, 68). This system paved the way for examining a broad range of PPIs, including both homodimeric and heterodimeric interactions (20, 35).

##### Recent advances

Several variations of the λ-B2H system have been developed to allow researchers to tailor the assay for different experimental needs (13, 19, 20, 35, 67, 68). For example, by modifying the promoter elements or altering the fusion constructs, the system can accommodate a range of interaction strengths and protein types, including those with weak or transient interactions (13, 35). This flexibility gives the system adaptability for HTP screening of protein libraries, facilitating the discovery of novel interactions and mapping interactomes (14, 19, 20, 68). Joung et al. (22) enhanced the λ-B2H system by replacing the *lacZ* reporter with HIS3 and implementing stringent selection conditions, which reduced endogenous protein interference and improved screening specificity (22, 23). In general, the “bait” protein is fused to the λ cI repressor, and the “prey” protein is fused to the RNA polymerase (RNAP) alpha subunit (13–15, 19). Upon interaction between the bait and prey proteins, the cI repressor and RNA polymerase α subunit are brought into close proximity. This allows RNA polymerase to bind the promoter and initiate transcription, activating a reporter gene such as *lacZ* (encoding β-galactosidase) or a gene conferring antibiotic resistance.

##### Summary

λ-B2H offers a versatile tool for PPIs, and its development marked a critical milestone, paving the way for subsequent innovations in two-hybrid methodologies.

### LexA-based B2H (LexA-B2H)

Dimitrova et al. (38) developed a LexA-B2H system that introduced a hybrid operator with two distinct half-sites, enabling repression of reporter gene expression only when heterodimers form (38). This was one of the first systems to genetically enforce heterodimer specificity, appearing around the same time as the dual-operator λ/434 variant described by Hays et al. (19). In addition to improving selectivity, the LexA system enabled better control over FP expression levels via flexible use of high- and low-copy plasmids, improving detection of stoichiometry-sensitive interactions (15, 38, 70, 71).

#### Basic setup

In LexA-B2H (Fig. 2B), wild-type and mutant LexA DBDs are fused to POIs to assess their interactions (14, 15, 37, 38). In the OFF state, POIs do not interact, preventing the LexA DBDs from binding to their respective operator half-sites and allowing transcription of the reporter gene to proceed (15, 38). In the ON state, the POIs interact, stabilizing the LexA binding at the hybrid operator and repressing reporter gene expression (15, 37, 38).

#### More detail

This system utilizes two compatible plasmids, one encoding a wild-type LexA DBD fusion and the other a mutant LexA DBD fusion. The mutant LexA variant is engineered to recognize a distinct mutated region of the operator sequence that occurs upstream of a wild-type region, ensuring selective detection of heterodimeric interactions (37). To facilitate in-frame fusion, the system includes six expression plasmids, three with ColE1 (high-copy) and three with p15A (low-copy) origins, each featuring unique multiple cloning sites in different reading frames. This design supports flexible cloning and ensures appropriate expression of fusion constructs (37). A key innovation of the LexA system is that the copy number can be strategically varied between the two fusion constructs, enabling better control over expression levels. This is particularly useful when one protein strongly homodimerizes, as expressing it from a low-copy vector can minimize self-association and thereby favor heterodimer formation. Reciprocal configurations (e.g., swapping the POIs between high- and low-copy vectors) allow investigators to confirm that observed repression results from heterodimerization rather than artifacts of overexpression or FP imbalance (37).

##### Example studies

LexA-B2H has been widely used to investigate PPIs in bacterial regulatory networks and enzymatic complexes. Daines and Silver (71) applied the LexA-B2H system to examine interactions among proteins involved in polysialic acid biosynthesis in *E. coli* K1, fusing NeuA, NeuB, NeuC, and NeuD to the LexA-DBD to assess dimerization (71). Their results confirmed NeuD as the strongest self-associating protein of the group, consistent with its known trimerization tendency, while also identifying a heterodimeric interaction between NeuB and NeuD. This demonstrated the system’s sensitivity in detecting both homodimeric and heterodimeric associations (71).

Daigle et al. (70) used LexA-B2H to characterize the interaction between MexR, a repressor of the mexAB-oprM multidrug efflux operon in *Pseudomonas aeruginosa*, and its modulator PA3719. This interaction was further validated using ITC, confirming a strong binding affinity. By demonstrating the regulatory effects of PPIs on antibiotic resistance, this study highlighted the utility of LexA-B2H for dissecting bacterial transcriptional networks and understanding multidrug efflux regulation.

##### Summary

LexA-B2H improves specificity for heterodimer detection through the use of an asymmetric operator and tunable expression via copy number control.

### AraC-B2H

Kornacker et al. (69) introduced a B2H system based on the AraC regulator (AraC-B2H), which uses interaction-dependent DNA looping to repress gene expression (13, 15, 69). AraC-B2H links PPIs to conformational changes in DNA architecture, creating a spatially regulated repression mechanism (14, 15, 69).

#### Basic setup

The AraC system (Fig. 2C) fuses the DBD of AraC and LexA to the two POIs (14, 15, 69). In the ON state, the absence of an interaction mediated by the fused POIs prevents DNA looping and allows the recruitment of RNA polymerase, resulting in reporter gene transcription. In the OFF state, an interaction between POIs facilitates the assembly of the AraC-LexA hybrid complex. This interaction promotes DNA looping and inhibits reporter gene transcription (14, 15, 69).

#### More detail

The AraC system was developed from the natural regulatory function of the AraC protein, which controls gene expression within the *E. coli* L-arabinose operon (14, 15, 69). A distinctive feature of the AraC system is its reliance on DNA looping. In the presence of *E. coli* integration host factor (IHF), a DNA-bending protein, AraC-mediated loops are stabilized, repressing transcriptional regulation (14, 15, 69). AraC binds cooperatively to two operator half-sites, I1 and I2, which are located upstream of the araBAD promoter (69). The I1 site has a higher affinity for AraC, and once bound, it facilitates the cooperative binding of another AraC subunit to I2, which is positioned closer to the promoter. The distance between these sites directly influences transcriptional activation. Modifying this spacing affects the strength of the reporter gene signal, allowing fine-tuning of system sensitivity. This flexibility enables the detection of both strong and weak PPIs (69).

##### Example studies

While this system has not been extensively utilized, Kornacker validated the system using model interactions, such as the LZ domains of Jun and Fos and the interaction of E6 with E6AP, where E6 refers to the E6 oncoprotein from human papillomavirus and E6AP is the E6-associated protein involved in ubiquitin-mediated proteolysis (69). These applications highlighted the system’s ability to detect interactions involving a wide range of dimerization domains (69). Furthermore, its integration with other molecular biology techniques has facilitated mapping of interaction domains, understanding regulatory networks, and identifying interaction inhibitors (14, 15, 69).

##### Technical considerations

It is important to note that the efficiency of AraC-B2H relies heavily on DNA looping efficiency. The formation and stability of these loops are influenced by the arrangement and spacing of operator sites and the involvement of auxiliary DNA-bending proteins such as IHF, which enhance loop stability (14, 15, 69). Proper tuning of these parameters is essential to ensure accurate and reproducible results.

##### Recent advances/application

Despite its demonstrated potential in detecting enzyme-substrate interactions *in vivo*, this system has not been adopted in subsequent research. The lack of further development may be attributed to challenges related to its scalability, sensitivity, or integration with other detection technologies. One possible reason for its limited use could be challenges in optimizing looping efficiency, ensuring consistent heterodimerization, or applying it in more diverse experimental contexts, such as large-scale screening assays. Another consideration is that alternative B2H systems, such as BacterioMatch and split adenylate cyclase, have emerged as more practical platforms for protein interaction screening.

##### Summary

The AraC-B2H tunable DNA looping mechanism enables spatially regulated repression, offering a unique approach to controllable gene expression in bacteria.

### Variants using transcription activation or enhancement

The following B2H variants utilize activation or enhancement of a reporter gene to detect a PPI. These three variant names are colored blue in Fig. 2.

### RNA Polymerase-B2H

Hochschild and Dove (54) introduced the RNAP-based B2H (RNAP-B2H) system, which links PPIs to reporter activation by directly recruiting RNA polymerase subunits fused to POIs (14, 15, 54).

#### Basic setup

The RNAP system (Fig. 2D) directly links PPIs to transcription by recruiting RNAP to its promoter (14, 15, 54). A repressor DBD is fused to POI-X, while POI-Y is fused to an RNAP subunit, typically α or ω. In the LOW state, the λ cI DBD fusion binds to its operator site, but without an interaction between the POIs, RNAP recruitment remains weak, resulting in minimal transcription. In contrast, the ON state occurs when the POIs interact, stabilizing RNAP at the promoter and enhancing transcription of the reporter gene (14, 15, 54).

#### More detail

The α and ω subunits of RNAP are commonly used in this system because they provide accessible surfaces for protein interaction without disrupting core transcriptional functions (15, 54). The α subunit contains two domains: the N-terminal domain (α-NTD) and the C-terminal domain (α-CTD). The α-NTD is responsible for RNAP assembly, while the α-CTD interacts with promoter elements and transcriptional regulators. In the RNAP-B2H system, the α-NTD is typically used for fusion, allowing the recruitment of RNAP through an engineered PPI. The ω subunit serves as an alternative fusion site and can enhance transcriptional output when used in tandem with α fusions (14, 15, 54). A key advantage is that bait-prey interactions directly trigger transcription without intermediate signaling steps. The cooperative nature of RNAP assembly allows even weak or transient interactions to stabilize subunit proximity and activate reporter expression, enabling the detection of low-affinity interactions (54).

##### Example studies

Hochschild and Dove used RNAP-B2H to examine interactions between yeast TFs Gal4 and Gal11P (54). Rao et al. (72) applied the RNAP-B2H system to identify regulators of transcription in *Chlamydia trachomatis*, an organism with limited genetic tools (72). Using a λCI-β-flap fusion as bait and screening a *Chlamydia* genomic library fused to the α-NTD of RNAP, they discovered CT663, a previously uncharacterized protein that directly interacts with both the β subunit and region 4 of the σ^66^ factor (72).

##### Technical considerations

Although RNAP-B2H provides distinct advantages, it also presents system-specific technical challenges, particularly concerning RNA polymerase adaptability. Unlike other B2H variants, in which FPs typically interact with DNA-binding elements or regulatory proteins, RNAP-B2H requires direct fusion to subunits of the RNA polymerase complex itself. This potentially introduces greater susceptibility to steric hindrance and folding constraints (23). While minimizing steric interference and preserving native interactions are important across all B2H platforms, these design factors may be especially critical in RNAP-B2H. In most systems, POIs are fused to regulatory proteins, such as λ cI, that influence transcription indirectly, without influencing RNA polymerase assembly. In contrast, RNAP-B2H involves direct fusion of a POI to an RNA polymerase subunit, making the proper assembly and function of the polymerase dependent on the integrity of the fusion construct (23). The ω subunit of RNAP, once considered dispensable, is now known to support RNAP stability, β′ folding, and stress-responsive transcription through the stringent response (73–77). Fusion of ω in systems like RNAP-B2H may disrupt these native functions, so constructs should use C-terminal tags with flexible linkers and be validated for RNAP integrity and stress responsiveness. This can be assessed by comparing reporter activity and core subunit expression in tagged versus untagged strains, along with growth under stress conditions.

##### Recent advances

More recent RNAP-B2H studies have extended applications to explore bacterial virulence mechanisms, regulatory pathways, and small-molecule interaction inhibitors. This technique was used to map interactions between Severe acute respiratory syndrome coronavirus 2 viral proteins and host factors, providing critical insights into the virus’s replication machinery and pathogenic mechanisms (34). These findings were instrumental in identifying potential therapeutic targets, showcasing the system’s relevance in addressing urgent global health challenges. The introduction of an alternate reporter gene has further expanded the system’s scope (14, 15, 34). Some adaptations of RNAP-B2H, which use fluorescent reporters, such as green fluorescent protein, have enabled real-time monitoring of interaction dynamics, improving the detection of transient and weak interactions by providing high-resolution insights into binding kinetics and temporal regulation (34). Such advances make the system particularly useful for studying complex regulatory networks and enzyme-substrate interactions (14, 15, 34). While this enhancement has proven useful in the RNAP-B2H system context, similar adaptations are possible in other B2H platforms.

The BacterioMatch II system, a variant of the RNAP method, has been developed by Agilent Technologies. It provides a commercially standardized platform for B2H studies (33). It employs dual reporters, HIS3 and *aadA*, which enable both nutritional and antibiotic-based selection to validate PPIs. The system’s use of low-copy plasmids (pBT and pTRG) and stringent selection with 3-amino-1,2,4-triazole minimizes background activation and improves discrimination between weak and strong interactors. While based on the classical λ cI and RNAP α-NTD fusion design, which may introduce false positives due to self-association, this is mitigated through provided self-activation controls and flexible vector design. The kit includes all necessary reagents including bait and prey vectors, competent cells, and detailed protocols, making it well-suited for both hypothesis-driven and HTP screening efforts (33).

##### Summary

RNAP-B2H amplifies weak or transient interaction signals through direct RNA polymerase recruitment, enabling sensitive detection of PPIs in complex systems.

### BACTH

Karimova et al. (49) developed the BACTH system, which detects PPIs by reconstituting adenylate cyclase activity in *E. coli cya⁻* strains (49). This design introduced cAMP signaling as an indirect readout, enabling detection of interactions throughout the cytoplasm and membranes (49).

#### Basic setup

The BACTH system (Fig. 2E) utilizes the catalytic domain of adenylate cyclase, divided into two fragments (T25 and T18), each fused to POIs (14, 49). When no interaction is present, the system remains in the OFF state. T25 and T18 remain separate, preventing adenylate cyclase reconstitution. As a result, cAMP synthesis does not occur, and the catabolite activator protein (CAP) remains inactive, preventing reporter gene expression. In the ON state, an interaction between POIs brings T25 and T18 into proximity, allowing functional complementation of adenylate cyclase. This reconstitution triggers cAMP synthesis, activating CAP, which then binds to the CAP binding site, leading to expression of the reporter gene (14, 49).

#### More detail

The BACTH method is unique in that PPIs can occur throughout the cell, as it does not rely on direct coupling to the transcriptional machinery. It specifically uses the catalytically inactive fragments *of Bordetella pertussis* adenylate cyclase: T25 (amino acids [aa] 1–224) and T18 (aa 225–399) (14, 49). These fragments do not spontaneously reassemble in the bacterial cytoplasm, ensuring that cAMP production and subsequent transcriptional activation occur only when an interaction brings them together, reducing background noise and increasing assay specificity. Similar to LexA, BACTH employs two compatible plasmids with differing copy numbers to independently express the T25 and T18 FP, enabling modulation of expression levels. This design optimizes the system for detecting both strong and weak interactions by balancing sensitivity and background signal. BACTH is also uniquely suited for detecting interactions in various cellular compartments such as membrane-associated interactions (14, 49). This versatility makes it particularly useful for investigating bacterial regulatory networks and membrane-bound complexes that cannot be efficiently analyzed using nuclear-based systems such as Y2H.

##### Example studies

The foundational study by Karimova et al. demonstrated the versatility of BACTH by investigating LZ interactions and testing larger proteins such as tyrosyl tRNA synthetase. The results confirmed the system’s capacity to detect diverse PPIs, including interactions between eukaryotic proteins, within a bacterial host. Its design provided critical insights into protein interaction networks and set the stage for subsequent modifications that expanded its applicability (14, 15, 49). More recently, Ouellette et al. (44) used BACTH to analyze interactions between membrane proteins in *E. coli* by applying the system to integral and peripherally associated membrane complexes (44). Their study emphasized the system’s ability to detect interactions that occur within the cellular membrane, a domain often challenging for traditional two-hybrid assays (44).

##### Technical considerations

A particular challenge lies in the system’s dependency on the cAMP signaling pathway, which may be influenced by metabolic conditions and growth phase variations. These factors necessitate more precise experimental conditions, such as maintaining bacterial cultures at optimal densities and standardizing induction times for hybrid protein expression (43). BACTH variants designed to detect disulfide-dependent PPIs rely on efficient and precise targeting of FPs to the periplasm. However, the confined space and compartmental organization of the periplasm can hinder proper orientation or proximity of the T25 and T18 fragments, decreasing the efficiency of adenylate cyclase reconstitution and subsequent cAMP signaling (50).

##### Recent advances

There have been several innovative adaptations of BACTH. Choi et al. (78) developed a temperature-sensitive and non-canonical amino acid (ncAA)-controlled biological logic gate for BACTH (78), integrating environmental stimuli directly into the assay. The temperature-sensitive feature allows for the fine-tuning of protein interactions under specific thermal conditions, while the incorporation of ncAAs serves as a conditional control mechanism, enabling interaction detection only in the presence of a given ncAA. This reduces background activation and improves assay fidelity. These features make the system particularly useful for studying dynamic regulatory pathways sensitive to environmental fluctuations. By offering greater experimental control, this modification has opened new avenues for dissecting complex protein networks under otherwise elusive physiological contexts (78).

In the traditional BACTH system, T25 and T18 fragments are expressed in the reducing cytoplasm, which hinders proper folding of proteins requiring disulfide bonds. Houot et al. addressed this by fusing these fragments to periplasmic signal peptides (e.g., *ompA*), directing their export via the secretory pathway (50). This allows interactions to occur in the oxidizing periplasm, enabling correct disulfide bond formation. This was shown to be essential for detecting the interaction between GIII^CTX^ and TolA, both of which are periplasmic-facing and rely on disulfide-stabilized folding (50).

In another study, Wu et al. (39) introduced fluorescence-based reporters to further enhance the functionality of BACTH. By incorporating fluorophores such as tdTomato, they coupled the assay with flow cytometry, allowing for real-time, HTP analysis of PPIs (39). This adaptation facilitates quantitative analysis of interaction dynamics, detailed insights into binding kinetics, and the simultaneous screening of large libraries for novel interactors (39). Unlike traditional reporter systems reliant on endpoint assays, the integration of flow cytometry ensures continuous monitoring of interactions. These innovations will be particularly effective for identifying subtle or transient interactions that might be missed in conventional PPI detection setups.

##### Summary

BACTH enables cAMP-based detection of PPIs in various subcellular contexts, including membrane-associated proteins, making it a versatile tool for studying diverse interaction types in *E. coli*.

### NGB2H

The NGB2H system is an extension of the BACTH approach, and so will be described in the same section. Developed by Boldridge et al. (29), NGB2H builds on BACTH by integrating DNA barcoding and NGS to enable scalable, quantitative PPI screening (29). Each bait-prey pair is linked to a unique barcode embedded in a fluorescent reporter, allowing precise measurement of interaction strength across thousands of protein pairs in parallel (29). This variation is covered in detail because it offers a much-needed innovation in B2H system detection, addressing the overreliance on β-galactosidase readouts.

#### Basic setup

NGB2H utilizes the same core mechanism as BACTH (Fig. 2E), relying on interaction-dependent reconstitution of adenylate cyclase to initiate transcription (29). However, it replaces traditional enzymatic reporters with a fluorescence-based readout linked to unique DNA barcodes. Upon PPI-mediated reconstitution, cAMP production activates cAMP receptor protein (CRP), driving expression of superfolder GFP. A synthetic DNA barcode embedded in the 3′ untranslated region (UTR) of the reporter is co-transcribed and enables quantification of interaction strength via RNA-seq (29).

#### More detail

NGB2H enables parallel synthesis and screening of diverse protein libraries, supporting systematic PPI mapping at scale (29). Each bait-prey pair is assigned multiple unique barcodes during cloning (Fig. S1). These are mapped to their respective constructs through early sequencing, when barcodes and coding regions are physically linked. This strategy allows confident tracking of interactions, even among homologous sequences, and eliminates the need for colony picking (29). By integrating DNA synthesis, barcode mapping, and inducible expression, NGB2H facilitates the detection of polymorphic PPIs, family-specific interactions, and rationally designed orthogonal sets. The TK310 background strain enhances the system’s dynamic range by enabling linear cAMP accumulation, outperforming β-galactosidase-based systems (29). Interaction strength is quantified by calculating the natural log of the median RNA-to-DNA read ratio for each barcode. This dual normalization accounts for variation in plasmid abundance, expression, and sequencing depth, ensuring reproducible measurements across thousands of protein pairs (29). Using multiple barcodes per construct improves robustness and minimizes bias. The system reliably detects low-affinity interactions, achieving over 100-fold dynamic range and replicate Pearson correlations above 0.98 (29).

#### Technical considerations

Barcode fidelity is critical to NGB2H performance. Misassignment during early mapping or sequencing errors can introduce false positives or negatives, though stringent quality control, such as read thresholds and barcode clustering, helps mitigate these risks (29). The reliance on short-read Illumina sequencing limits barcode and construct complexity, though long-read platforms may overcome this in future versions (29). The system is currently optimized for binary PPIs and does not resolve orientation-specific interactions or higher-order complexes (29). Because expression is measured relative to other constructs in the same pool, comparing scores across libraries requires internal standards (29).

NGB2H was validated using synthetic libraries of orthogonal coiled-coil (CC) proteins, screening over 26,000 interactions across CCNG1 and CCmax libraries (29). This large-scale effort demonstrated the system’s scalability and reproducibility and led to iterative refinement of CC scoring models, resulting in the iCipa algorithm and the largest experimentally validated set of orthogonal CCs to date (29). Developed to overcome limitations of traditional B2H platforms, including poor scalability, low sensitivity, and labor-intensive formats, NGB2H replaces colony screening with programmed bait-prey combinations and barcode-based quantification. This enables systematic detection of low-affinity or transient interactions and supports advanced applications such as polymorphic landscape mapping, orthogonal pair design, and *de novo* interface modeling—capabilities not accessible with earlier two-hybrid systems.

#### Summary

NGB2H overcomes key limitations of traditional B2H systems by combining barcoded reporters, NGS-based quantification, and flexible library design. Its integration of synthetic biology and computational tools enables scalable interaction mapping, orthogonal module development, and new applications in protein engineering and synthetic circuit design (78, 79).

### ToxR-B2H

Dziejman and Mekalanos (30) introduced the ToxR-B2H system, which detects PPIs by enhancing reporter gene transcription through dimerization of ToxR FPs within the native lipid bilayer of *E. coli* (30, 31, 58).

#### Basic setup

The ToxR-B2H system (Fig. 2F) is designed to monitor interactions between transmembrane helices through a transcriptional enhancement mechanism (30, 32, 80). In the LOW state, the fused transmembrane helices POIs do not interact, preventing ToxR dimerization and reducing its ability to bind the *ctx* promoter. The reporter genes are expressed at a very low level, preventing bacterial growth under selective conditions. In the ON state, interaction among the POIs, fused between the transmembrane helices, facilitates ToxR dimerization, allowing cooperative binding to its promoter. This enhances the expression of the reporter gene, enabling bacterial survival in selective media (30, 32, 80).

#### More detail

The ToxR protein consists of a cytoplasmic DBD, a transmembrane region, and a periplasmic domain. In this system, the DBD of ToxR is fused to the transmembrane helices of interest, with the FP anchored in the bacterial inner membrane (30, 31). A major advantage of the ToxR system is its ability to assess membrane protein interactions without requiring detergent solubilization, which can disrupt native folding and assembly (31, 32). Thus, it is particularly valuable for studying membrane protein oligomerization and stability (80). The system’s sensitivity to helix-helix interactions also makes it useful for identifying residues critical for dimerization, as well as for screening libraries to discover novel interaction motifs (30, 32). By incorporating mutational analysis, such as alanine scanning, key residues involved in membrane protein assembly can be mapped (31).

##### Example studies

ToxR-B2H has been used to investigate the dimerization of glycophorin A’s transmembrane domain, revealing key residues that mediate helix association and demonstrating their functional relevance *in vivo* (31, 32). Additionally, the system has proven valuable for identifying mutations that disrupt interactions, with potential applications in understanding membrane protein pathogenesis and designing targeted therapies (30, 81).

##### Technical challenges

Interactions in the lipid bilayer environment may differ significantly from those observed in detergent micelles, highlighting the importance of maintaining physiological conditions during experiments (31, 32). Environmental factors such as pH, osmolarity, and temperature further influence ToxR transcriptional activity, complicating experimental reproducibility and requiring tightly controlled assay conditions (82).

##### Recent advances

Modifications to the ToxR system have expanded its application beyond simple transmembrane dimerization analysis. For example, it has been adapted for HTP screening, enabling the screening of synthetic transmembrane peptide libraries leading to the discovery of novel helix-helix interaction motifs under native membrane conditions (32). Hennecke et al. (83) further advanced the platform by reengineering the ToxR FP to study PPIs in both the periplasmic and cytoplasmic compartments of *E. coli* (83). By replacing the native ToxR transmembrane segment POIs, they demonstrated that the system could detect asymmetric and compartment-specific interactions, including those involving model proteins with weak or directionally biased dimerization.

##### Summary

ToxR-B2H enables native membrane-based detection of transmembrane protein interactions *in vivo*, offering a detergent-free approach for studying periplasmic and membrane-localized PPIs (83).

### Variants utilizing cell survival

The following B2H variants detect PPIs through their ability to rescue the cell under selective conditions. These two variant names are colored black in Fig. 2.

### Murine dihydrofolate reductase-based B2H (mDHFR-B2H)

Pelletier et al. (52) introduced the mDHFR-B2H system, which detects PPIs through interaction-dependent reconstitution of mDHFR enzyme activity, enabling bacterial survival in the presence of trimethoprim (14, 15, 51, 52). This transcription-independent system provides a functional readout based on restored metabolic activity rather than reporter gene activation.

#### Basic setup

The mDHFR system (Fig. 2G) consists of two POIs fused to the N-terminal (F[1,2]) and C-terminal (F[3]) fragments of mDHFR (14, 15, 52). In the OFF state, the F[1,2] and F[3] fragments of mDHFR remain separate, preventing enzyme reassembly. Without active DHFR, tetrahydrofolate synthesis is disrupted, and cells are unable to survive in media containing trimethoprim, a drug that selectively inhibits endogenous *E. coli* DHFR but does not affect mDHFR. In the ON state, interaction between the fused POIs facilitates reassembly of the mDHFR fragments, restoring enzymatic function. The restored mDHFR activity enables tetrahydrofolate synthesis, allowing bacteria to survive and grow (14, 15, 51, 52).

#### More detail

The reconstitution of enzyme activity can be detected via enzymatic assays that measure the reduction of dihydrofolate to tetrahydrofolate using substrates such as NADPH (14, 15, 51, 52). The system’s reliance on enzyme activity as a direct readout for PPIs bypasses transcriptional or translational readouts common to other B2H systems (51, 52). Its flexibility has been demonstrated through the use of an I114A mutation in the F[3] fragment, which destabilizes enzyme reassembly and increases selection pressure. This modification allows for the discrimination between strong and weak interactions by reducing the likelihood of spontaneous reassembly.

##### Example studies

Pelletier et al. (52) first demonstrated the application of the mDHFR-B2H system using the well-characterized LZ domains of Fos and Jun, which are known to interact (52). In their 1999 study, Pelletier et al. expanded on their B2H system by introducing a *cis*-complementation strategy to optimize PPIs through *in vivo* selection (51). They constructed combinatorial libraries of the *E. coli* DHFR fragments, systematically mutating residues near the fragment interface to generate variants with improved reassembly efficiency. These libraries were then screened in *E. coli*, with successful DHFR reconstitution conferring resistance to trimethoprim, allowing for the selection of mutations that enhanced fragment association.

To test the system’s ability to optimize interactions beyond DHFR, they applied it to the LZ interaction between Fos and Jun. They created combinatorial libraries of Jun mutants and screened for variants with increased affinity for Fos, identifying mutations that strengthened the interaction. This study demonstrated that the system could be used for directed evolution, selecting for optimized protein interactions *in vivo* based on functional complementation (51).

Despite its demonstrated utility, mDHFR-B2H remains a niche approach rather than a widely adopted screening tool. Several factors may have contributed to its limited use in recent years. One of the main challenges is this system’s reliance on enzymatic reassembly, which could make it less versatile for certain interaction studies, particularly those involving larger or structurally complex proteins (52). Similar to the AraC-based system, mDHFR-B2H may have been outcompeted by commercially available B2H platforms, which provide more streamlined workflows and greater compatibility with large-scale screening applications.

##### Technical challenges

An important consideration for this system is its dependence on structural reassembly to restore enzymatic activity. While proper FP folding is important in all two-hybrid systems, the mDHFR system may be uniquely stringent because the reconstitution of catalytic function, not just proximity or partial complex formation, is the functional readout. As a result, optimizing linker length and expression conditions becomes critical to achieving high signal-to-noise and reducing background from improperly folded or inactive complexes. Substrate concentration and buffer composition should also be optimized when measuring NADPH-dependent reduction of dihydrofolate (51, 52). It has been noted that domains with strong dimerization or oligomerization tendencies, such as LZ, work best with this system (51, 52).

##### Summary

mDHFR-B2H provides a transcription-independent survival-based readout through enzymatic reconstitution, offering a functional alternative for PPI detection.

### Reverse-B2H

Horswill et al. (36) developed the RTHS, which flips the standard B2H design to detect interaction disruption rather than formation (36).

#### Basic setup

RTHS (Fig. 2F) modifies conventional B2H systems by coupling PPI disruption to a genetic selection readout, enabling the identification of small-molecule inhibitors or peptides that interfere with complex formation (36). In the ON state, disruption of the interaction by a small-molecule inhibitor or peptide prevents repressor complex formation, leading to the activation of reporter genes. The resulting expression of HIS3 and KanR rescues bacterial growth. In contrast, the OFF state occurs when the inhibitor does not bind to a fused POI. In this state, a repressor complex forms and binds to the operator region, blocking the expression of reporter genes. As a result, bacterial growth is inhibited under selective conditions (36).

#### More detail

This system integrates two core technologies: B2H selection and split-intein-mediated circular ligation of peptides and proteins (SICLOPPS), which facilitates the intracellular synthesis of cyclic peptide libraries for screening (36). SICLOPPS technology enhances screening capabilities by generating genetically encoded cyclic peptide libraries, which are used to identify inhibitors with constrained structures that improve target binding and stability. Within this setup, each plasmid encodes a cyclic peptide variant, as well as two FPs encoding a fragment of the mR2 repressor fused to a POI. When a cyclic peptide disrupts a PPI being monitored by RTHS, repression of reporter gene expression is relieved, enabling bacterial growth under selective conditions. Coupling peptide activity to cell survival allows HTP screening of libraries (∼10⁸ variants) for rare inhibitors. Hits can be readily identified by sequencing the plasmid inserts (36).

A primary advantage of RTHS is that screening occurs *in vivo*. This means that inhibitors must function in a biologically relevant context where factors such as endogenous protein interactions, molecular crowding, and cellular metabolism influence their activity. Because of this, molecules identified using RTHS are more likely to be functionally effective *in vivo*. In contrast, affinity-based *in vitro* assays may identify molecules that bind to a target but fail to disrupt its function within a cellular environment (36). By linking bacterial survival to interaction disruption, RTHS ensures that only inhibitors capable of modulating PPIs in a physiologically relevant setting are selected.

##### Example studies

Horswill et al*.* demonstrated this system’s effectiveness using the well-characterized protein complexes, FKBP12-rapamycin-associated protein and HIV-1 protease. Their results underscored the system’s capacity for HTP screening and its utility in uncovering unique modes of action for small molecules, including cyclic peptide inhibitors generated via SICLOPPS (36). To further validate the system’s efficacy, they applied it to ribonucleotide reductase (RR), a heterodimeric enzyme essential for DNA synthesis. Using SICLOPPS-derived cyclic peptides, they identified inhibitors that prevented RR subunit association, demonstrating the system’s ability to isolate functional modulators of biologically relevant PPIs (36).

##### Technical challenges

The cyclic peptides must be expressed and processed efficiently *in vivo*, as incomplete intein splicing can reduce library integrity (36). Additionally, high library complexity requires robust transformation efficiency to achieve adequate coverage. Another limitation is that not all peptides can fold into conformations capable of penetrating interaction interfaces, which may bias screening results toward certain structural classes. Finally, the reverse two-hybrid configuration depends on strong basal repression, so background expression or leaky reporters can complicate the interpretation of weak disruptors (36, 84).

Kjelstrup et al. (84) utilized the RTHS framework to identify cyclic peptides that inhibit dimerization of the *Staphylococcus aureus* β-sliding clamp (DnaN), an essential replication protein (84). Using the SICLOPPS method to produce cyclic peptides intracellularly, they screened for inhibitors that disrupted DnaN-DnaN interactions. Disruption was linked to pyrF repression, enabling selection on 5-fluoroorotic acid (84). Selected peptides were shown to inhibit DNA replication, induce SOS response, and cause cell death in *S. aureus*, confirming their functional relevance in the native host (84).

##### Summary

RTHS enables *in vivo* selection of PPI inhibitors by coupling interaction disruption to cell death, enriching for peptides that functionally disrupt complexes in a biologically relevant context.

## OTHER COMMON PPI METHODS AND POTENTIAL FOR INTEGRATION WITH B2H

For comparative analysis, and because combinatorial approaches are key to obtaining a complete picture of protein interactions (2, 4–6, 85), the next section will provide an overview of the most commonly employed PPI detection methods, highlighting their significance and potential for integration with B2H systems.

The study of PPIs has progressed alongside advances in molecular biology and genomics. Early observations, such as trypsin-inhibitor binding and antibody interactions with plant toxins, date back to the late 19th century, although the recognition that both components were proteins came later (5, 86, 87). The emergence of molecular biology in the mid-20th century laid the groundwork for systematic PPI studies, leading to innovations such as phage display and Y2H screening (9, 86, 88). By the 1990s, genomics enabled large-scale mapping of interaction networks, and the availability of complete genome sequences revealed evolutionary trends in PPIs (5, 86).

PPI detection methods generally fall into two categories: HTP approaches for global network screening and lower-throughput (LTP) methods for focused, high-confidence validation. HTP techniques support systems-level analysis, while LTP methods, including co-IP and biochemical reconstitution, enable mechanistic insights into specific interactions (5). Due to the breadth of this topic, the following sections will focus on commonly used methods for detecting PPIs, specifically those for discovery and confirmation. Techniques primarily related to structural determination, kinetic analysis, or detailed characterization of PPIs are beyond the scope of this review. For a more comprehensive discussion on PPI detection strategies, readers may find the work of Titeca et al. (6), Walport et al. (1), Tsuchiya et al. (89), and Xian et al. (90) particularly insightful (1, 6, 90). A summary of common PPI detection methods and their applications is provided in Table 2.

**TABLE 2:** Strengths and weaknesses of common PPI detection methods

Method	Applications	Advantages	Disadvantages
Affinity purification			
Co-immunoprecipitation (co-IP)	Validation of direct interactions; enrichment of complexes	Straightforward design; compatible with downstream analysis (e.g., MS)	Often low-throughput; may miss weak or transient interactions; tagging required
Pull-down assays			
Biophysical quantification and thermodynamics			
Surface plasmon resonance (SPR)	Quantify binding strength, stoichiometry, or size of complexes	Quantitative; low false positives; compatible with purified proteins; real-time detection possible with SPR and MST	Often require large sample amounts or purified proteins; susceptible to artifacts from buffer conditions or immobilization; requires specialized equipment
Isothermal Titration Calorimetry (ITC)			
Microscale thermophoresis (MST)			
Analytical ultracentrifugation (AUC)			
Size-exclusion chromatography (SEC)			
Microscopy			
FRET	Real-time interaction monitoring in live cells.	High sensitivity and spatial resolution	Not ideal for detecting weak, indirect, or long-range interactions; require specialized imaging equipment and conditions
Bioluminescence resonance energy transfer (BRET)			
Split-luciferase complementation assay			
Proximity labeling			
Proximity-dependent biotin identification (BioID)	Proximal interactions in living cells	Captures transient and weak interactions; operates in native cellular context	Potential for labeling non-specific nearby proteins; requires optimization of biotinylation conditions
Mass spectrometry			
AP-MS	Complex identification, interface mapping, architecture of protein networks	Can resolve large complexes; identifies both direct and indirect partners	Data analysis can be complex; sensitive to sample prep quality; prone to false positives in cross-linking; can miss transient or weak interactions
Cross-link mass spectrometry (XL-MS)			
Tandem affinity purification (TAP) coupled with mass spectrometry			
Display and screening-based			
Phage display	High-throughput screening of protein interactions	Scalable; effective for epitope mapping	May not reflect physiological conditions; potential for non-specific interactions
Two-hybrid			
Y2H	High-throughput binary interaction screening *in vivo*	Efficient for detecting novel interactions.	High false-positive/negative rate; often confined to yeast nucleus; misses membrane-associated interactions

### Affinity purification

Pull-down and co-IP assays are widely accepted LTP biochemical techniques for detecting PPIs due to their reliability, simplicity, and ability to complement HTP methods (2, 3). Pull-down assays use affinity-tagged bait proteins, such as glutathione S-transferase or His-tags, immobilized on glutathione or nickel-nitrilotriacetic acid resin, to isolate binding partners from lysates. Bound proteins are eluted and analyzed using sodium dodecyl sulfate-polyacrylamide gel electrophoresis or mass spectrometry (MS). These assays are effective for validating direct, stable interactions *in vitro* (2), but may miss weak or transient interactions and can introduce non-physiological artifacts due to tagging (2, 3).

Co-IP assays attempt to preserve PPIs as they occur *in vivo* by isolating complexes from cell lysates without perturbation of endogenous expression levels or cellular signaling states (3). Target-specific antibodies are used to co-precipitate associated proteins from lysates, followed by immunoblotting or mass spectrometry (3). Co-IP is particularly useful for detecting interactions influenced by PTMs, making it useful for studying dynamic signaling pathways (2). Despite its strengths, results depend heavily on antibody specificity, and false positives can arise from indirect associations or cross-reactivity. Protein solubilization steps can often lead to the formation of membrane micelles which can disrupt native PPIs and promote artificial associations (91–93). Appropriate controls, such as isotype-matched antibodies and pre-clearing, are essential to ensure specificity (2, 3). Together, these methods offer complementary advantages for elucidating PPI networks and refining the functional understanding of complex biological systems (2, 3). Together, these assays provide complementary strengths for confirming PPIs and offer mechanistic insights into complex biological systems. Their proven utility also makes them suitable for validating interactions discovered through other platforms, including B2H (2, 3).

### Surface plasmon resonance (SPR)

SPR is a label-free optical technique widely utilized for studying PPIs. It detects binding events by measuring changes in refractive index when one interaction partner, immobilized on a gold-coated sensor chip, binds its counterpart in solution (1, 2). One interaction partner, immobilized on the sensor surface, binds its counterpart from the analyte solution, resulting in measurable shifts in the resonance angle of reflected light. In addition to confirming interactions, SPR can quantify binding affinities and provide kinetic parameters such as association and dissociation rates, offering detailed insight into complex stability and dynamics (1, 2).

SPR requires only small sample volumes and supports detection at nanomolar concentrations, making it particularly suitable for rare or difficult-to-purify proteins (2). It is also compatible with a wide range of biomolecular targets, including proteins, nucleic acids, and small molecules (2). SPR may introduce artifacts due to protein immobilization or surface effects, such as conformational changes in the immobilized protein or steric hindrance experienced by the analyte in close proximity to the sensor surface (1, 2). These issues can be mitigated by using site-specific immobilization strategies, such as engineered cysteine residues or biotin-streptavidin coupling (1, 2). SPR complements other techniques by enabling real-time interaction analysis and kinetic profiling, and it can support the validation of interactions identified through systems such as B2H.

### Microscopy

FRET and bioluminescence resonance energy transfer (BRET) are microscopy-based techniques that provide high spatial and temporal resolution for studying PPIs. FRET detects non-radiative energy transfer between donor and acceptor fluorophores in close proximity (1–10 nm), enabling real-time monitoring of interactions and conformational changes in live cells (1, 2, 6). Fluorescence lifetime imaging microscopy further enhances FRET sensitivity and precision (1). BRET uses a bioluminescent donor, such as luciferase, which eliminates the need for external excitation and reduces background autofluorescence (2, 6). Improvements in luciferase engineering have enhanced BRET’s sensitivity and signal stability, extending its use to HTP screening and tissue-level imaging (6).

Despite their strengths, these methods face limitations. FRET may suffer from photobleaching and spectral overlap, while BRET can be affected by steric hindrance during protein labeling (1, 2, 6). Both techniques require careful optimization to minimize artifacts and ensure reliable results. Wille et al. (94) demonstrated a versatile platform that combines B2H with techniques such as FRET, BRET, and split luciferase assays, using a modular set of plasmids based on Gateway cloning (94). Gateway cloning is a recombination-based method that enables rapid and site-specific transfer of DNA fragments between vectors. Wille’s system allows POIs to be easily fused to various detection tags, such as SNAP-tag, HaloTag, or Gaussia luciferase, and tested across multiple imaging or reporter-based assays. This streamlined approach minimizes cloning workload and supports simultaneous analysis of interaction dynamics and protein localization, offering a flexible platform where microscopy techniques can complement B2H assays (94).

### Proximity labeling

Proximity labeling techniques, including BioID (proximity-dependent biotin identification) and APEX (engineered ascorbate peroxidase), are widely used for detecting PPIs in live cells. These methods involve fusing an enzyme to a POI, which covalently labels nearby proteins within a defined radius (1, 6, 95). BioID uses a promiscuous biotin ligase (BirA*) to biotinylate proximal lysine residues, enabling enrichment and identification of interacting proteins by mass spectrometry. Although effective for capturing weak or transient interactions, its long labeling time limits temporal resolution. APEX utilizes engineered ascorbate peroxidase to catalyze rapid labeling through biotin-phenol oxidation in the presence of hydrogen peroxide, making it suited for dynamic or membrane-associated complexes (1, 6).

Both methods can produce non-specific labeling, and enzyme fusions may cause steric hindrance. Variants such as TurboID and miniTurbo have improved labeling efficiency and reduced enzyme size (1, 6). Olson et al. (95) combined B2H with APEX to study interactions at the *Chlamydia trachomatis* vacuole membrane. B2H assays identified a range of Inc (inclusion membrane) proteins that interact with host factors involved in vesicle trafficking, including SNARE proteins and Rab GTPases. Subsequent APEX2 labeling in infected cells validated spatial proximity between these host targets and inclusion-localized Inc proteins, providing complementary evidence for functional host-pathogen interactions at the vacuolar interface (95).

### Mass spectrometry

MS is a sensitive, HTP method widely used to detect PPIs in complex biological systems. Among various MS-based strategies, AP-MS is the most common (3, 5, 6). In this approach, a tagged POI is purified from lysed cells along with its interacting partners, which are then identified by MS. AP-MS is particularly valuable for mapping interactions in native environments, including mammalian systems (4–6). However, it cannot readily differentiate between direct and indirect interactions, which complicates data interpretation. AP-MS cannot easily distinguish between direct and indirect interactions, and purification steps may introduce false positives or miss transient associations (3–5). Tandem affinity purification MS improves specificity by using dual tags and sequential purification steps, though it still struggles with transient or weak interactions (3–6).

Cross-linking MS (XL-MS) helps overcome these limitations by capturing spatial constraints between proteins (2, 5, 6). A recent study by Stahl et al. (96) combined XL-MS with AlphaFold2-based deep learning to predict protein interaction interfaces within the *Bacillus subtilis* proteome. B2H assays were used to validate a subset of the structurally inferred interactions, including a previously uncharacterized interaction between the iron homeostasis regulator Fur and the transcriptional repressor YdiH. This functionally validated interaction supported the proposed model of an iron-responsive regulatory complex, illustrating how B2H can provide *in vivo* support for structurally predicted PPIs (96).

## FUTURE DIRECTIONS FOR B2H

The field of PPI research is poised to make significant advances, driven by emerging methodologies and integrative approaches. Traditional methods have provided foundational insights, but they often rely on expensive equipment, specialized training, and complex data analysis (2, 5, 15). As global resource centers grow in number and accessibility, new kits and user-friendly software are expected to democratize these technologies, enabling even inexperienced researchers to prepare samples and collaborate with advanced facilities for detailed evaluations of PPIs (2). This shift will likely transform many state-of-the-art methods into routine assays, opening avenues for broader applications.

Incorporating two-hybrid technologies with complementary approaches, such as MS and targeted mutagenesis, will enable the construction of detailed PPI maps and reveal cross-talk between proteins across diverse cellular processes (5, 14). Advances in precision medicine will benefit from high-quality PPI networks that can identify combinations of driver mutations responsible for disease progression (4, 97, 98). Integrating PPI data with other omics approaches—such as transcriptomics, proteomics, and metabolomics—will be essential for interpreting genotype-phenotype relationships and uncovering mechanisms of disease at a systems biology level (4, 14, 97, 99, 100). Constructing tissue-specific, developmentally contextual, or dynamically regulated PPI networks will be particularly valuable for drug discovery and biomarker identification (4, 99, 100).

As proteomics evolves, the goal is to precisely define the PPI landscape, including spatial distribution, interaction affinity, and kinetic behavior. This will require integrating data from diverse techniques and prioritizing methods that avoid the need for tags, labeling, or immobilization (5, 85). Future analytical methods will likely draw on untapped principles in physics and chemistry to measure interaction dynamics *in situ* and in real-time, extending the capabilities of current technologies (85). With thoughtful implementation, B2H systems can complement advanced technologies and support PPI discovery through scalable and accessible platforms.

## SUPPLEMENTAL MATERIAL

Figure S1 jb.00129-25-s0001.docx NGB2H detection analysis overview.

ASM does not own the copyrights to Supplemental Material that may be linked to, or accessed through, an article. The authors have granted ASM a non-exclusive, world-wide license to publish the Supplemental Material files. Please contact the corresponding author directly for reuse.
