# Supplementary material for: Bacterial two-hybrid systems evolved: innovations for protein-protein interaction research
Source: J Bacteriol. 2025 Aug 1;207(8):e00129-25. doi: 10.1128/jb.00129-25 (PMC12369361; doi:10.1128/jb.00129-25)
Supplement: Figure S1 — NGB2H detection analysis overview. [file jb.00129-25-s0001.docx]

**
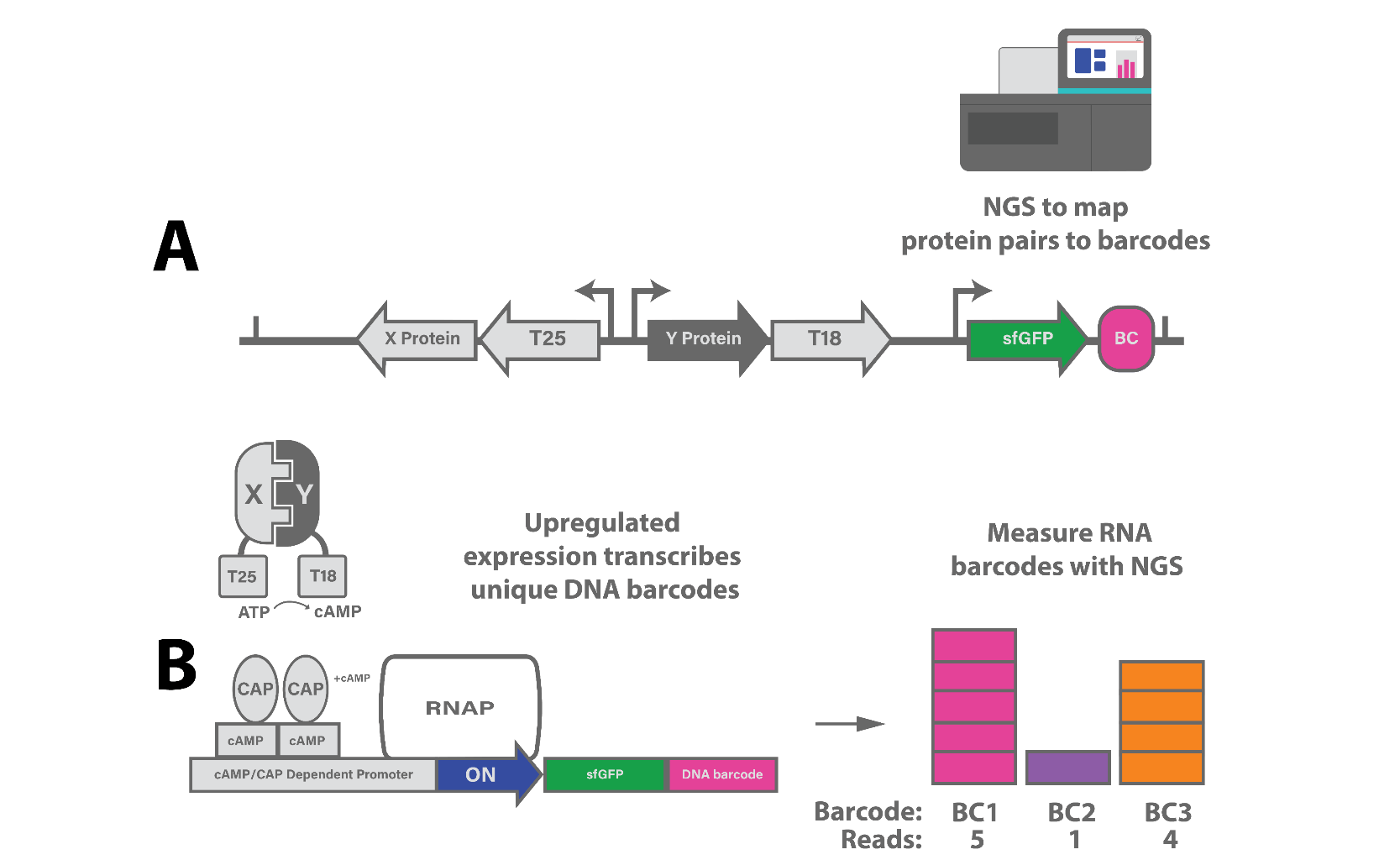
SUPPLEMENTAL MATERIALS**

**Figure S1: NGB2H Detection Analysis Overview.** (A) Genes encoding hybrid proteins (T25–X and T18–Y) are expressed from divergent, inducible promoters on a shared plasmid. Each interaction pair is linked to a unique 20-bp DNA barcode and mapped using NGS. (B) If the POIs interact, they reconstitute adenylate cyclase, producing cAMP, which activates transcription of a reporter gene encoding sfGFP and the DNA barcode. Interaction strength is quantified by sequencing the RNA barcodes and normalizing to plasmid abundance via DNA barcode counts. RNA and DNA are extracted in parallel and analyzed in a single NGS run to calculate interaction scores.
